# Comparison of proton pump inhibitors and histamine 2 receptor antagonists for stress ulcer prophylaxis in the intensive care unit

**DOI:** 10.1038/s41598-021-98069-7

**Published:** 2021-09-16

**Authors:** Myung Jin Song, Seok Kim, Dachung Boo, Changhyun Park, Sooyoung Yoo, Ho Il Yoon, Young-Jae Cho

**Affiliations:** 1 Division of Pulmonary and Critical Care Medicine, Department of Internal Medicine, Seoul National University College of Medicine, Seoul National University Bundang Hospital, 82 Gumi-ro, 173 Beon-gil, Bundang-gu, Seongnam-si, Gyeonggi-do 13620 Republic of Korea; 2 Office of eHealth Research and Business, Seoul National University Bundang Hospital, Seongnam, Republic of Korea

**Keywords:** Outcomes research, Upper gastrointestinal bleeding

## Abstract

Proton pump inhibitors (PPIs), followed by histamine 2 receptor antagonists (H2RAs), are the most commonly used drugs to prevent gastrointestinal bleeding in critically ill patients through stress ulcer prophylaxis. The relative efficacy and drug-related adverse events of PPIs and H2RAs remain unclear. In this retrospective, observational, comparative cohort study, PPIs and H2RAs for stress ulcer prophylaxis in critically ill patients were compared using a common data model. After propensity matching, 935 patients from each treatment group (PPI or H2RA) were selected. The PPI group had a significantly higher 90-day mortality than the H2RA group (relative risk: 1.28; *P* = 0.01). However, no significant inter-group differences in the risk of clinically important gastrointestinal bleeding were observed. Moreover, there were no significant differences between the groups concerning the risk of pneumonia or *Clostridioides difficile* infection, which are known potential adverse events related to these drugs. Subgroup analysis of patients with high disease severity were consistent with those of the total propensity score-matched population. These findings do not support the current recommendations, which prefer PPIs for gastrointestinal bleeding prophylaxis in the intensive care unit.

## Introduction

Critically ill patients are at risk of stress ulcer-related gastrointestinal tract (GI) bleeding; which is known to affect between 2.6 and 6.6% of these patients, being associated with 2- to 4-times higher risk of death^1–3^. The pathophysiology of stress ulcers in critically ill patients is not fully understood; however, they are believed to be related to the disruption of mucosal protective defenses against gastric acid, gastric mucosal hypoperfusion, increased acid production, and oxidative injury to the digestive tract^4^.

Proton pump inhibitors (PPIs) are the most commonly used drugs in intensive care units (ICUs) to prevent GI bleeding caused by stress ulcers, followed by histamine 2 receptor antagonists (H2RAs)^5^. Stress ulcer prophylaxis (SUP) using either type of drug reduces the risk of clinically important GI bleeding. Nonetheless, concerns about adverse events associated with these drugs, including pneumonia and *Clostridioides difficile* infection, have been raised, with some studies suggesting that these risks peak shortly after starting the drugs, which is the case of most patients in the ICU^6–11^.

The current guidelines for SUP in the ICU recommend the use of either PPI or H2RA for patients who have risk factors for GI bleeding^12–14^. However, the preferred agents vary among guidelines^12–14^. Several randomized controlled trials (RCTs) have been conducted to compare PPIs and H2RAs for SUP^15–20^. In the most recently published network meta-analysis, the evidence for the effects of PPIs and H2RAs on the mortality rate and GI bleeding lacked robustness^21^. Considering that over half of the patients in the ICU are prescribed drugs for SUP, further research to answer this uncertainty is essential^22^.

The Observational Medical Outcomes Partnership (OMOP) common data model (CDM) is a logical and semantic information model for health care data that can translate diverse data sources into a standardized format^23,24^. Heterogeneous data sources can be integrated using a CDM-based vocabulary, thus allowing the analysis of large-scale data in clinical studies. Observational Health Data Sciences and Informatics (OHDSI) is an open-science community that has created a robust library of open-source analytical tools to support OMOP CDM^25^.

This large retrospective cohort study aimed to compare PPIs and H2RAs for SUP in patients in the ICU using the OHDSI available tools for OMOP CDM.

## Results

### Baseline characteristics of the study cohort

**Figure 1:**
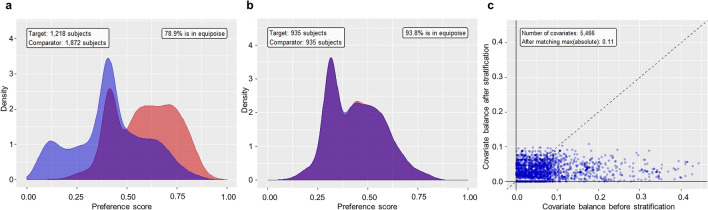
Diagnostics of propensity score matching performance. (**a**) Distribution of preference scores before propensity score matching. (**b**) Distribution of preference scores after propensity score matching. (**c**) Distribution of standardized mean differences in the means of individual covariates before and after stratification by propensity score.

A total of 3330 patients were included in the analysis (1338 and 1992 in the PPI and H2RA groups, respectively). After propensity score matching, 935 patients from each group were selected. The preference score, a transformed propensity score that adjusts for differences in the sizes of the two treatment groups, was highly overlapped after propensity score matching, indicating that the two groups were well balanced (Fig. 1a,b). The standardized mean differences of all covariates before and after stratification by propensity score are shown in Fig. 1c, and the baseline characteristics of the study population before and after propensity score matching are shown in Table 1. After matching, the mean age was 67 years in both groups, and women comprised 37.1% and 35.8% of the PPI and H2RA groups, respectively. The mean SUP treatment period during 90-day hospitalization was 19.35 days in the PPI group and 17.00 days in the H2RA group.

**Table 1:** Baseline characteristics of the study population before and after propensity score matching.

Characteristic	Before matching			After matching		
	PPI (n = 1338)	H2RA (n = 1992)	SMD^a^	PPI (n = 935)	H2RA (n = 935)	SMD^a^
Age, mean (year)	68	64	0.10	67	67	0.00
Gender, female (%)	442 (36.3%)	753 (40.2%)	− 0.08	347 (37.1%)	335 (35.8%)	0.03
**Medical history**						
General						
Chronic liver disease	15 (1.2%)	7 (0.4%)	0.10	10 (1.1%)	7 (0.7%)	0.03
Chronic obstructive lung disease	43 (3.5%)	37 (2.0%)	0.10	34 (3.6%)	31 (3.3%)	0.02
Diabetes mellitus	91 (7.5%)	145 (7.7%)	0.01	64 (6.8%)	60 (6.4%)	0.02
Hypertension	39 (3.2%)	72 (3.8%)	0.03	29 (3.1%)	28 (3.0%)	0.01
Chronic kidney disease	51 (4.2%)	44 (2.4%)	0.10	37 (4.0%)	32 (3.4%)	0.03
Acute kidney injury	12 (1.0%)	14 (0.7%)	0.03	6 (0.6%)	12 (1.3%)	0.07
Cerebrovascular disease	131 (10.8%)	178 (9.5%)	0.04	104 (11.1%)	90 (9.6%)	0.05
Ischemic heart disease	67 (5.5%)	170 (9.1%)	0.14	49 (5.2%)	44 (4.7%)	0.02
Neoplasm						
Hematologic neoplasm	18 (1.5%)	3 (0.2%)	0.15	9 (1.0%)	3 (0.3%)	0.08
Malignant lymphoma	28 (2.3%)	10 (0.5%)	0.15	15 (1.6%)	10 (1.1%)	0.05
Malignant neoplasm of anorectum	15 (1.2%)	16 (0.9%)	0.04	9 (1.0%)	13 (1.4%)	0.04
Malignant tumor of breast	11 (0.9%)	5 (0.3%)	0.08	8 (0.9%)	4 (0.4%)	0.05
Malignant tumor of colon	25 (2.1%)	32 (1.7%)	0.03	12 (1.3%)	29 (3.1%)	0.12
Malignant tumor of lung	85 (7.0%)	101 (5.4%)	0.07	63 (6.7%)	73 (7.8%)	0.04
Malignant tumor of urinary bladder	8 (0.7%)	17 (0.9%)	0.03	5 (0.5%)	15 (1.6%)	0.10
Primary malignant neoplasm of prostate	7 (0.6%)	6 (0.3%)	0.04	4 (0.4%)	5 (0.5%)	0.02
**Medication use during ICU admission**						
Steroids	515 (42.3%)	779 (41.6%)	0.01	382 (40.9%)	381 (40.7%)	0.00
Anticoagulants	569 (46.7%)	1071 (57.2%)	0.21	431 (46.1%)	453 (48.4%)	0.05

*H2RA* histamine-2 receptor antagonist, *PPI* proton pump inhibitor, *SMD* standardized mean difference.

^a^Standardized mean difference less than 0.1 indicates good balance of the characteristics after propensity score matching.

### Primary outcome

Of the 935 patients in each group, 242 (25.9%) died in the PPI group and 204 (21.8%) died in the H2RA group by day 90 in the hospital. This 28% higher risk of 90-day in-hospital mortality was statistically significant (relative risk [RR] 1.28; 95% confidence interval [CI] 1.07–1.55; *P* = 0.01) (Table 2). The results for the primary outcome were consistent when analyzed after trimming the propensity score to include those between the 2.5th and 97.5th percentiles (RR, 1.29; 95% CI 1.06–1.57; *P* = 0.01) (Supplementary Table S1).

**Table 2:** Risk of primary and secondary outcomes.

Total propensity score matched population				
	PPI (n = 935)	H2RA (n = 935)	Relative risk (95% CI)	*P* value
**Primary outcome**				
90-day in-hospital mortality	242 (25.9%)	204 (21.8%)	1.28 (1.07–1.55)	0.01
**Secondary outcomes**				
Efficacy of stress ulcer prophylaxis				
Gastrointestinal tract bleeding	15 (1.6%)	16 (1.7%)	1.01 (0.50–2.06)	0.97
Drug-related adverse events				
*Clostridium difficile* infection	4 (0.4%)	3 (0.3%)	1.47 (0.32–7.44)	0.63
Pneumonia	89 (9.5%)	89 (9.5%)	1.08 (0.81–1.45)	0.59

Subgroup analysis (APACHE II ≥ 25)				
	PPI (n = 444)	H2RA (n = 444)	Relative risk (95% CI)	*P* value
**Primary outcome**				
90-day in-hospital mortality	139 (31.3%)	112 (25.2%)	1.31 (1.02–1.68)	0.03
**Secondary outcomes**				
Efficacy of stress ulcer prophylaxis				
Gastrointestinal tract bleeding	7 (1.6%)	10 (2.6%)	0.73 (0.26–1.89)	0.52
Drug related complications				
* Clostridium difficile* infection	1 (0.2%)	3 (0.7%)	0.36 (0.02–2.80)	0.43
Pneumonia	42 (9.5%)	48 (10.8%)	0.92 (0.61–1.39)	0.70

*APACHE* acute physiologic assessment and chronic health evaluation, *CI* confidence interval, *H2RA* histamine-2 receptor antagonist, *PPI* proton pump inhibitor.

### Secondary outcomes

There was no significant difference between the two groups in the risk of clinically important GI bleeding, which occurred in 1.6% of the PPI group and 1.7% of the H2RA group (RR 1.01; 95% CI 0.50–2.06; *P* = 0.97). There were also no significant differences between the PPI and H2RA groups concerning the risk of pneumonia (RR 1.08; 95% CI 0.81–1.45; *P* = 0.59) and *C. difficile* infection (RR 1.47; 95% CI 0.32–7.44; *P* = 0.63), which are known potential adverse events related to these drugs (Table 2).

### Subgroup analysis

Subgroup analysis was conducted in a high disease severity group including patients with Acute Physiologic Assessment and Chronic Health Evaluation (APACHE) II scores ≥ 25 (Table 2). The results for the primary and secondary outcomes were consistent with those of the total propensity score-matched population. Importantly, 90-day in-hospital mortality was significantly higher in the PPI group (by 31%) than in the H2RA group (RR 1.31; 95% CI 1.02–1.68; *P* = 0.03). The risk of clinically important GI bleeding (RR 0.73; 95% CI 0.26–1.89; *P* = 0.52), pneumonia (RR 0.92; 95% CI 0.61–1.39; *P* = 0.70), and *C. difficile* infection (RR 0.36; 95% CI 0.02–2.80; *P* = 0.43) were not significantly different between the groups.

## Discussion

In this retrospective cohort study using CDM, we showed that using PPIs for SUP increases the risk of mortality compared with H2RAs, despite no significant differences in clinically important GI bleeding, pneumonia, and *C. difficile* infection between the groups were observed. The results for the outcomes were consistent in a subgroup analysis of patients with APACHE II scores ≥ 25, which indicates high disease severity.

Previous RCT studies and meta-analyses comparing drugs for SUP could not reflect real-world practice and the extremely heterogenous characteristics of patients in the ICU^15–20^. Moreover, the traditional method of reviewing medical records to obtain real-world data is labor-intensive and prone to human error^26^. In this study, we analyzed a total of 3330 patients and > 16 years of medical records, which would be virtually impossible without CDM analysis. This approach allowed to review a vast amount of real-world data captured during routine clinical care. Furthermore, our research findings confirm the applicability of CDM analysis to the ICU and suggest the possibility of future CDM-based distributed network research using ICU data^25^.

Recently, two large international RCTs raised concerns that using PPIs for SUP may increase the risk of mortality^20,22^. Our study also showed that PPIs are associated with increased mortality in patients in the ICU. The exact mechanism underlying this association is not clear. A possible explanation is that PPIs may increase the risk of infection through immune modulation, as they have been reported to impair the immune system by inhibiting polymorphonuclear neutrophil, cytotoxic T lymphocyte, and natural killer cell activity^22,27,28^. Additionally, excessive stomach alkalinity induced by acid suppression could result in insufficient eradication of ingested pathogens, with alterations in various immunomodulatory and anti-inflammatory effects^29,30^. However, we evaluated the incidence of pneumonia and *C. difficile* infection to estimate the susceptibility to infection and found no differences between the two groups. Notably, only new-onset pneumonia in the ICU was detected using the diagnostic codes in this study; therefore, the effects of the drugs on pneumonia progression could not be assessed. As most ICU admissions in this study were due to pneumonia, this is a significant limitation in evaluating consequent susceptibility to pneumonia. For *C. difficile* infection, the observed incidence was lower in this study than in a previous observational report^31^. To increase the accuracy of *C. difficile* infection detection, we analyzed not only the diagnostic codes, but also the diagnostic test results and medication codes. However, it remains possible that some cases of *C. difficile* infection went undiagnosed. Further studies are needed to confirm the clinical impact of the experimentally observed immunomodulatory effects of PPIs. Without a clear mechanistic explanation, the potential effects of PPIs on mortality should be interpreted with caution.

Our results showed that the incidence of clinically important GI bleeding did not differ between PPI and H2RA treatments, in both the total propensity score-matched population and high disease severity subgroup. The demonstration that PPIs were superior to H2RAs in ulcer prevention and recovery in non-critically ill patients led many clinicians to expect benefits from PPIs rather than H2RAs in critically ill patients as well^1,28^. Nonetheless, our results do not support this hypothesis or the results of a recent systematic review, which reported that PPIs have benefits over H2RAs in preventing clinically important GI bleeding in high-risk patients, although with low-certainty evidence^21^. Our findings indicate the need to reconsider the practice of preferentially prescribing PPIs for SUP in the ICU.

This study has several limitations. First, only data from index hospitalizations were included; therefore, the mortality rate may be an underestimate. Second, as we measured GI bleeding events based on hemoglobin levels and required transfusions without assessing their clinical significance, overall bleeding events may have been under- or overestimated. However, the incidence of clinically important GI bleeding event in this study was comparable with its incidence in the recent pragmatic RCT^20^. Third, as discussed above, the incidence of pneumonia and *C. difficile* infection may also have been underestimated. Fourth, as the risk factors for SUP were not subdivided, we could not identify subgroups that could particularly benefit from PPI treatment.

In conclusion, the present study provides real-world evidence on SUP in the ICU using CDM. PPIs were associated with increased mortality compared with H2RAs, whereas the occurrences of clinically important GI bleeding, pneumonia, and *C. difficile* infection were not significantly different between the groups. Our findings suggest that intensivists should be aware of the potential increased risks of mortality with PPIs and provide proper guidance regarding their usage, considering the widespread use of these agents.

## Methods

### Study design and data sources

This was a retrospective, observational, comparative cohort study that used electronic health record data on > 1.8 million patients treated at Seoul National University Bundang Hospital (SNUBH), a tertiary university hospital in a metropolitan area. Data were transformed into the OMOP CDM, version 5.3. As the data sources were de-identified, this study was approved with a waiver of informed consent by the institutional review board of SNUBH (IRB No: X-2102-666-902). All methods were performed in accordance with the relevant guidelines of the institutional review board of SNUBH.

### Study population and exposure

**Figure 2:**
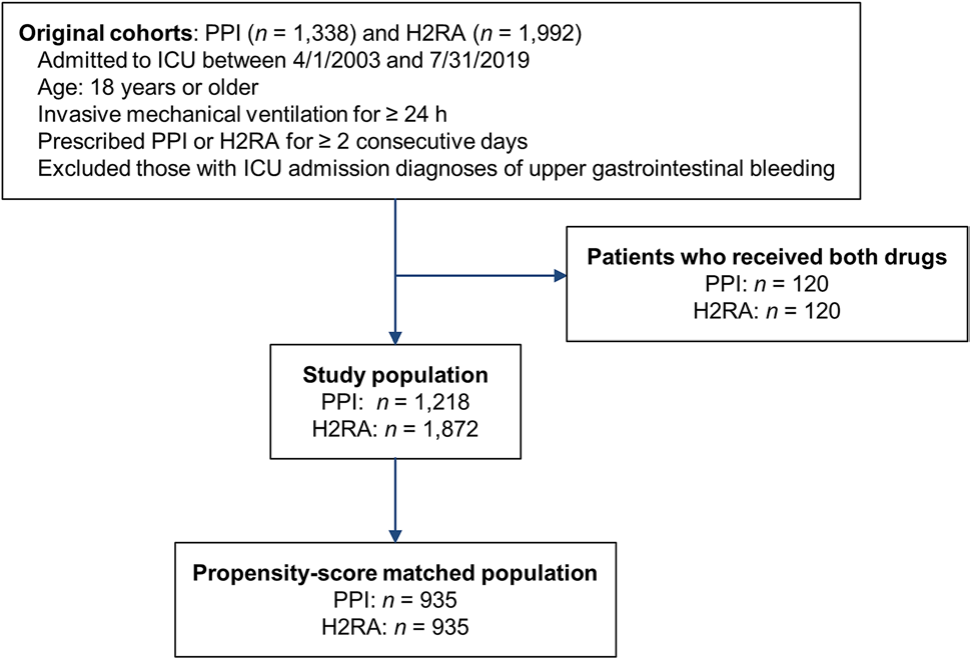
Attrition diagram of the study population. *H2RA* histamine 2 receptor antagonist, *PPI* proton pump inhibitor.

**Figure 3:**
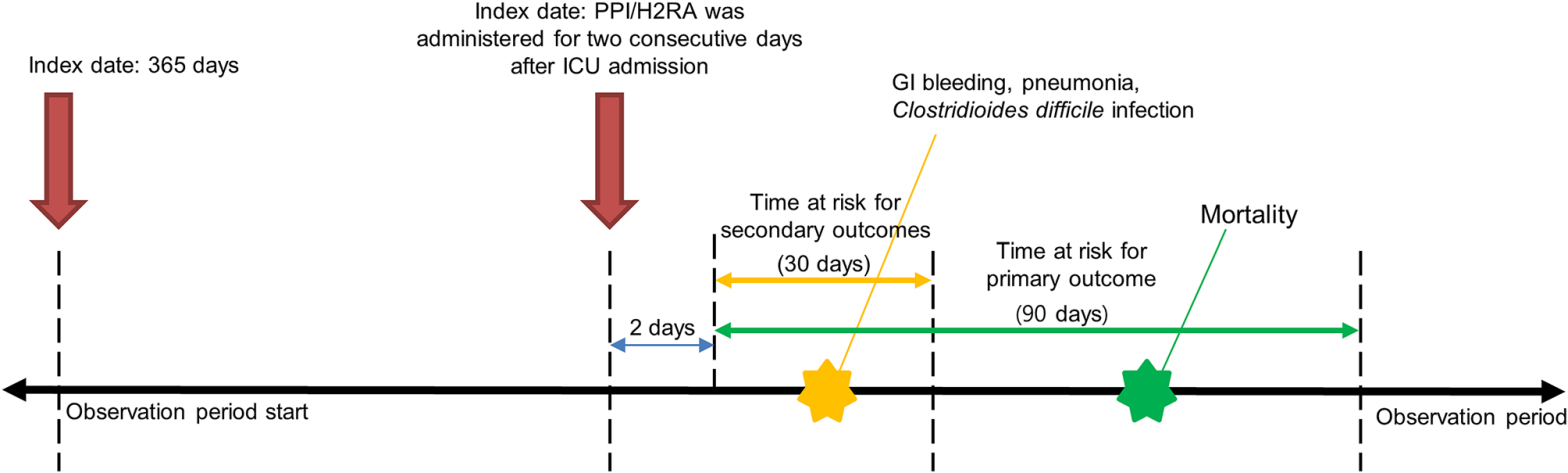
Study design. *H2RA* histamine 2 receptor antagonist, *GI* gastrointestinal tract, *PPI* proton pump inhibitor.

We identified adult patients aged ≥ 18 years admitted to the ICU who required invasive mechanical ventilation for ≥ 24 h between April 1, 2003, and July 31, 2019. Patients who were prescribed PPIs or H2RAs for ≥ 2 consecutive days after ICU admission were enrolled. The following patients were excluded from the study: (1) those with an ICU admission diagnosis of upper GI bleeding, (2) those who experienced GI bleeding within 48 h of the index date, and (3) those prescribed both PPIs and H2RAs during their ICU admission (Fig. 2). We defined the index date as the day PPIs or H2RAs had been administered in the ICU for 2 days. Outcome events that occurred 48 h after the index date were measured. The observation period started 365 days before the index date and ended on the last visit of the patient to the hospital or the date of death (Fig. 3).

### Outcomes

The primary outcome was in-hospital all-cause mortality up to 90 days after the index date. As the benefit obtained through SUP and the harm from drug-related adverse events are opposing endpoints, mortality was set as the primary endpoint, which can combine composite endpoints. The secondary outcomes were clinically important GI bleeding, pneumonia, and *C. difficile* infection. Clinically important GI bleeding was defined as a diagnosis of GI hemorrhage with decrease in hemoglobin ≥ 20 g/L or the transfusion of two or more units of packed red blood cells. Pneumonia was defined according to Tenth Revision of the International Classification of Diseases (ICD-10) codes [nosocomial pneumonia (J189), other pneumonia, organism unspecified (J188), other viral pneumonia (J1288), other bacterial pneumonia (J158), healthcare-associated pneumonia (J189), and ventilator-associated pneumonia (J677)]. *C. difficile* infection was defined as a diagnosis based on the ICD-10 code, presence of pseudomembranous colitis, positive *C. difficile* toxin assay results, or prescription of antibiotics for *C. difficile* infection (oral vancomycin or metronidazole).

### Statistical analysis

We used the population level estimation methodology and analysis tools provided by OHDSI. Propensity score matching was performed to relieve imbalances between the target and comparator cohorts of the original study populations, which have the potential for selection bias owing to the retrospective observational nature of the study. The propensity score is the probability that a patient will receive targeted treatment based on covariates from the start of observational period until the index date. By matching target and comparator patients with similar propensity scores, we constructed a balanced cohort.

Over 5000 variables were selected as baseline covariates, including demographics (sex, age, index year, and month), conditions (diagnosis, chief complaint), medications, procedures, measurements (laboratory results, vital signs, echocardiograms, transthoracic echocardiography, among others), observations (past history, family history, and nursing care records), Charlson comorbidity index, and CHADS_2_ score. Variables to be predicted according to the target and comparator were excluded. Covariates used in the study are shown in Supplementary Table S2.

Propensity scores were computed using a large-scale registered logistic regression model fitted by tenfold cross validation using the Cyclops package from OHDSI. To build matched cohorts, we performed 1:1 propensity score matching using a caliper of 0.2-times the standard deviation of the logit. The Cox proportional hazard model was applied to estimate the hazard ratios of outcomes, along with the associated 95% confidence interval (CI) and nominal *P* value. Two tailed *P* < 0.05 was considered statistically significant.

## Supplementary Information

Supplementary Information 1.

Supplementary Information 2.

## Data Availability

CDM data are designed to support a distributed research network. Thus, access to the data is restricted on internal private networks and are not publicly available.

## Abbreviations

CDM: Common data model
CI: Confidence interval
ICU: Intensive care units
OHDSI: Observational Health Data Sciences and Informatics
OMOP: Observational Medical Outcomes Partnership
PPI: Proton pump inhibitor
RCT: Randomized controlled trials
RR: Relative risk
SUP: Stress ulcer prophylaxis
