# Supplementary material for: Comparison of proton pump inhibitors and histamine 2 receptor antagonists for stress ulcer prophylaxis in the intensive care unit
Source: Sci Rep. 2021 Sep 16;11:18467. doi: 10.1038/s41598-021-98069-7 (PMC8446063; doi:10.1038/s41598-021-98069-7)
Supplement: Supplementary file 1 — Supplementary Information 1. [file 41598_2021_98069_MOESM1_ESM.docx]

*Supplementary information*

**Comparison of proton pump inhibitors and histamine 2 receptor antagonists for stress ulcer prophylaxis in the intensive care unit**

Myung Jin Song^1^, Seok Kim^2^, Dachung Boo^2^, Changhyun Park^2^, Sooyoung Yoo^2^, Ho Il Yoon^1^, Young-Jae Cho^1^

^1^Division of Pulmonary and Critical Care Medicine, Department of Internal Medicine, Seoul National University College of Medicine, Seoul National University Bundang Hospital, Seongnam, Republic of Korea

^2^Office of eHealth Research and Business, Seoul National University Bundang Hospital, Seongnam, Republic of Korea

**Corresponding author:** Young-Jae Cho, M.D., M.P.H., Ph.D.

Division of Pulmonary and Critical Care Medicine, Department of Internal Medicine, Seoul National University College of Medicine, Seoul National University Bundang Hospital, 82 Gumi-ro, 173 Beon-gil, Bundang-gu, Seongnam-si, Gyeonggi-do 13620, Republic of Korea

Fax: +82.31-787-4050; Tel: +82-31-787-7058

E-mail address: lungdrcho@snubh.org; [lungdrcho@gmail.com](mailto:lungdrcho@gmail.com)

**Supplementary Table S1.** Outcome risk after trimming propensity scores to include those between the 2.5^th^ and 97.5^th^ percentiles

| **Total propensity score matched population** | | | | | |
| --- | --- | --- | --- | --- | --- |
| Outcome | | PPI (n=859) | H2RA (n=859) | Relative risk (95% CI) | *P*-value |
| **Primary outcome** | |  |  |  |  |
|  | 90-day in-hospital mortality | 214 (24.9%) | 181 (21.1%) | 1.29 (1.06–1.57) | 0.01 |
| **Secondary outcomes** | |  |  |  |  |
| Efficacy of stress ulcer prophylaxis | | |  |  |  |
|  | Gastrointestinal tract bleeding | 13 (1.5%) | 16 (1.9%) | 0.88 (0.42–1.83) | 0.74 |
| Drug-related adverse events | |  |  |  |  |
|  | Clostridium difficile infection | 3 (3.5%) | 3 (0.3%) | 1.10 (0.20–5.96) | 0.91 |
|  | Pneumonia | 79 (9.2%) | 85 (9.9%) | 1.01 (0.74–1.38) | 0.93 |
| **Subgroup analysis (APACHE II ≥ 25)** | | | | | |
| Outcome | | PPI (n=438) | H2RA (n=438) | Relative risk (95% CI) | *P*-value |
| **Primary outcome** | |  |  |  |  |
|  | 90-day in-hospital mortality | 135 (30.8%) | 111 (25.3%) | 1.29 (1.00–1.66) | 0.05 |
| **Secondary outcomes** | |  |  |  |  |
| Efficacy of stress ulcer prophylaxis | | |  |  |  |
|  | Gastrointestinal tract bleeding | 7 (1.6%) | 10 (2.3%) | 0.73(0.26–1.90) | 0.53 |
| Drug-related adverse events | |  |  |  |  |
|  | Clostridium difficile infection | 1 (0.2%) | 3 (0.7%) | 0.36(0.02–2.80) | 0.43 |
|  | Pneumonia | 41 (9.4%) | 48 (11.0%) | 0.90(0.59–1.37) | 0.63 |

APACHE, acute physiologic assessment and chronic health evaluation; CI, confidence interval; H2RA, histamine-2 receptor antagonist; PPI, proton pump inhibitor
